# Correction: A method for supervoxel-wise association studies of age and other non-imaging variables from coronary computed tomography angiograms

**DOI:** 10.1038/s41598-026-60195-5

**Published:** 2026-07-01

**Authors:** Johan Öfverstedt, Elin Lundström, Göran Bergström, Joel Kullberg, Håkan Ahlström

**Affiliations:** 1 Radiology, Department of Surgical Sciences, Uppsala University, Uppsala, Sweden; 2 Antaros Medical, Mölndal, Sweden; 3 Department of Molecular and Clinical Medicine, Institute of Medicine, Sahlgrenska Academy, University of Gothenburg, Gothenburg, Sweden; 4 Department of Clinical Physiology, Sahlgrenska University Hospital, Region Västra Götaland, Gotheburg, Sweden; 5 Department of Surgical Sciences, SciLifeLab, Uppsala University, Uppsala, Sweden

Correction to: *Scientific Reports* 10.1038/s41598-026-46350-y, published online 31 March 2026

The original version of this Article contained an error in Figure 10, where a watermark was inadvertently published over the Figure.

The original Figure 10 and accompanying legend appears below.

**Fig. 10:**
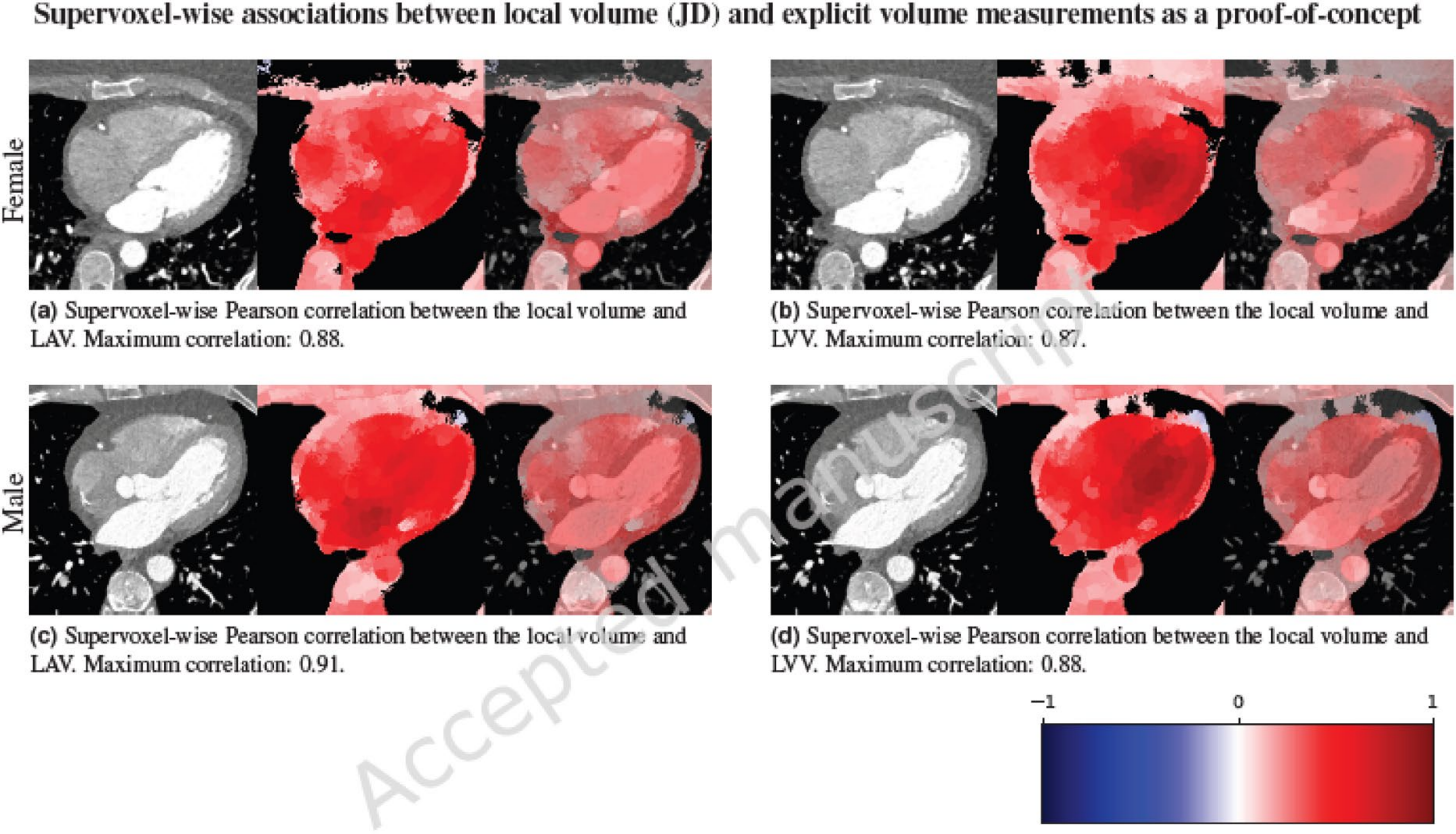
Supervoxel-wise results of Pearson correlation between JD and LAV/LVV, here illustrated on a single slice for each variable and sex. Each image is displayed as a reference image slice, correlation maps (where non-significance is shown as black), and the correlation map overlaid on the reference image. High maximum correlations between the JD and the corresponding explicit measurements are detected within both the LA and LV regions.

The original Article has been corrected.

