# Supplementary material for: A method for supervoxel-wise association studies of age and other non-imaging variables from coronary computed tomography angiograms
Source: Sci Rep. 2026 Mar 31;16:11000. doi: 10.1038/s41598-026-46350-y (PMC13043740; doi:10.1038/s41598-026-46350-y)
Supplement: Supplementary file 1 — Supplementary Information. [file 41598_2026_46350_MOESM1_ESM.pdf]

A Additional Supervoxel-wise Association Maps

Here we include additional supervoxel-wise association maps for JD and age for female subjects (Fig. S-1, for JD and age for male subjects (Fig. S-2), for density and age for female subjects (Fig. S-3), and for density and age for male subjects (Fig. S-4).

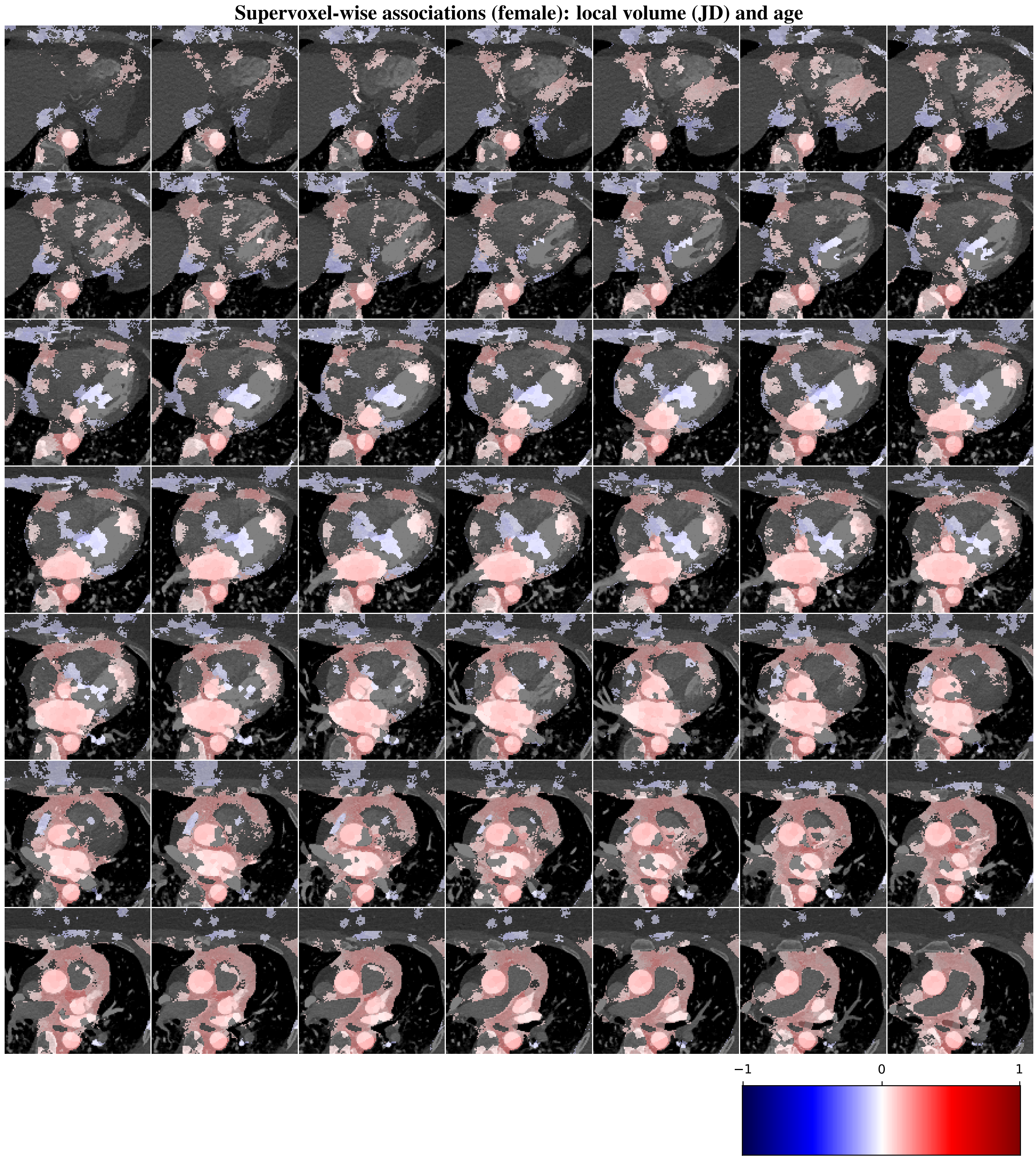

Figure S-1. Supervoxel-wise Pearson correlation for 49 evenly spaced axial slices, placed left to right, top to bottom.

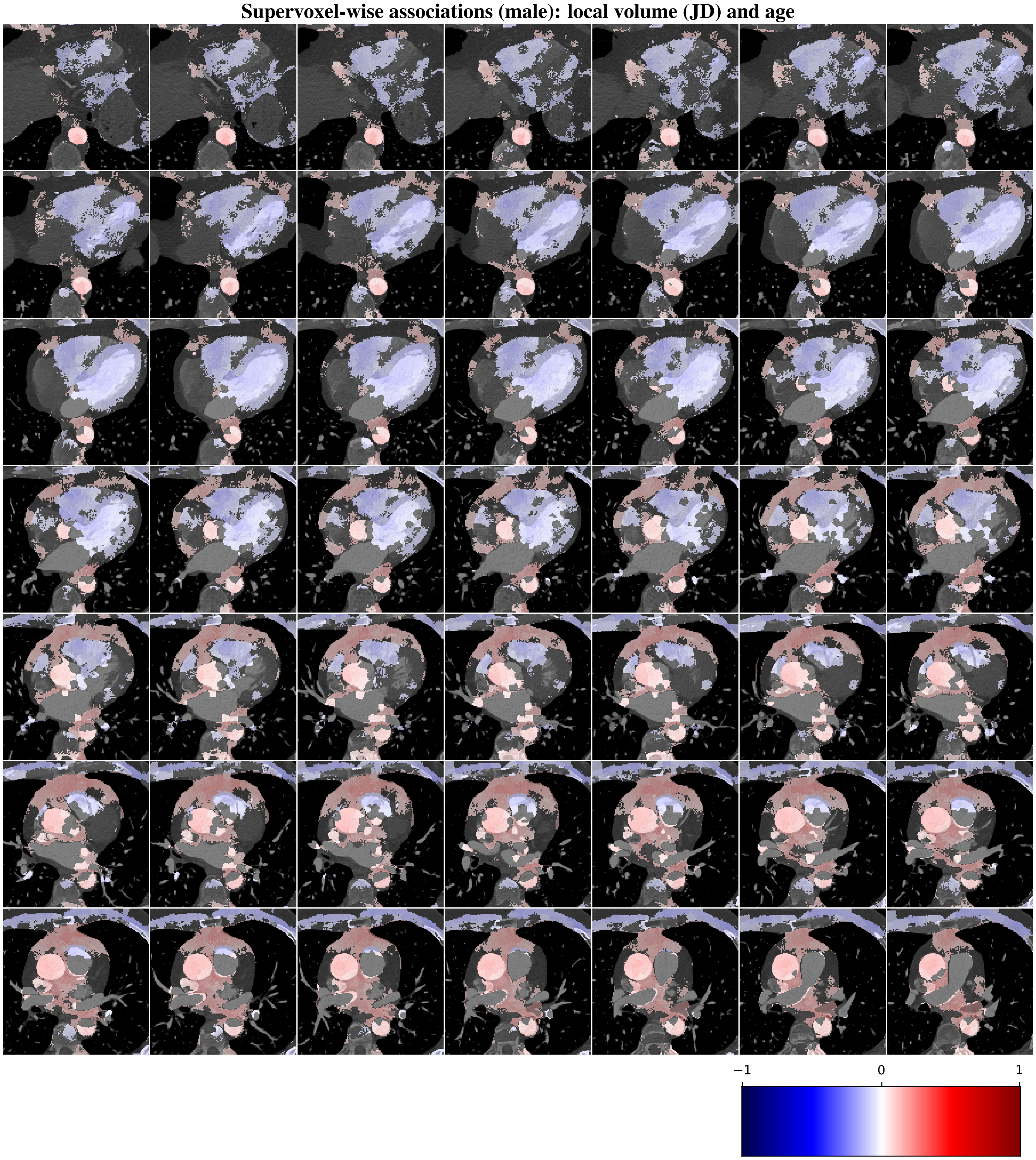

Figure S-2. Supervoxel-wise Pearson correlation for 49 evenly spaced axial slices, placed left to right, top to bottom.

Supervoxel-wise associations (female): density (HU) and age

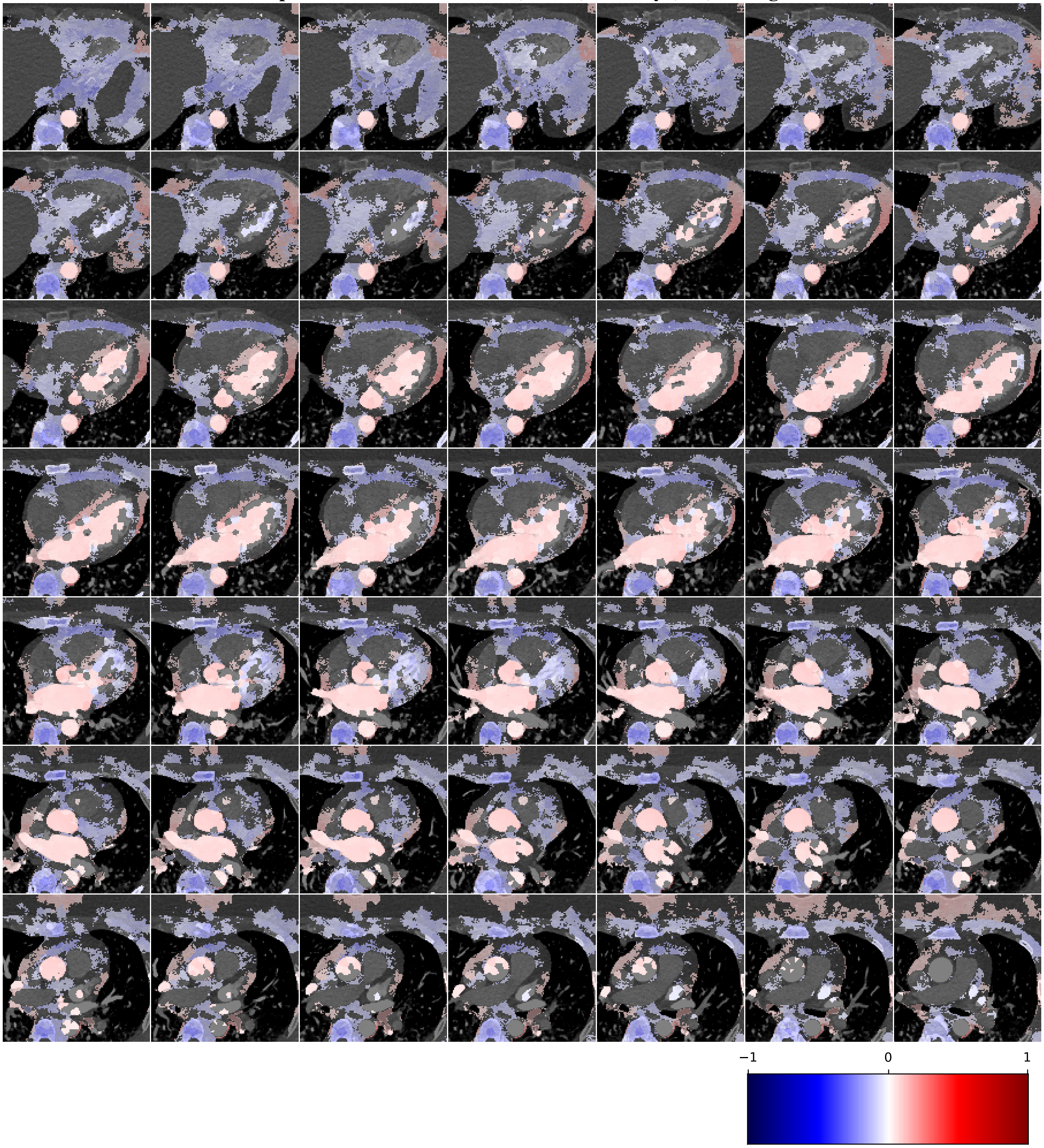

Figure S-3. Supervoxel-wise Pearson correlation for 49 evenly spaced axial slices, placed left to right, top to bottom.

Supervoxel-wise associations (male): density (HU) and age

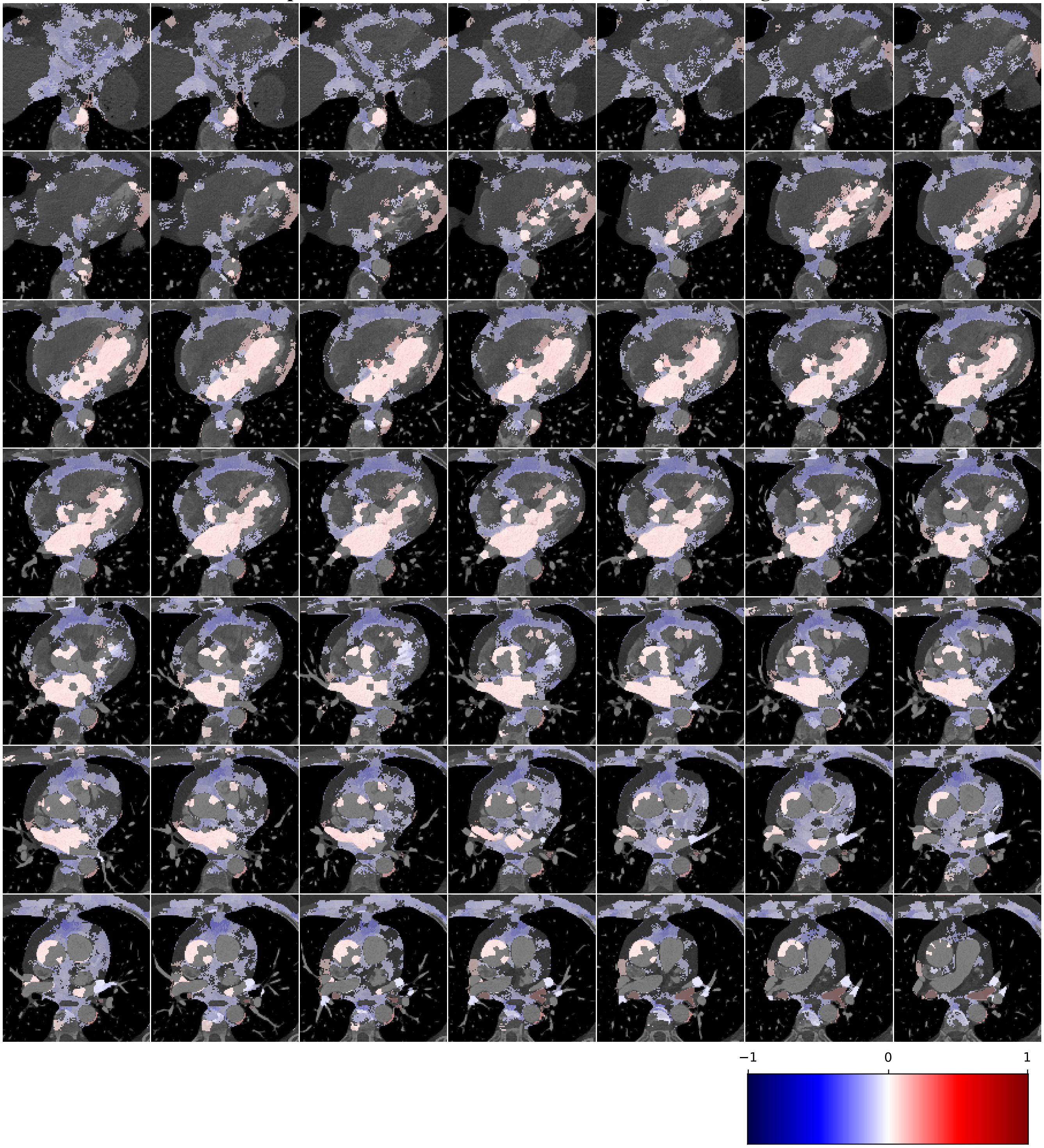

Figure S-4. Supervoxel-wise Pearson correlation for 49 evenly spaced axial slices, placed left to right, top to bottom.

B Additional Slices for Aggregate and Deviation Images/Jacobian Determinant Images from the Image Registration Process

In Fig. S-5, we show the registration results for two additional axial slices.

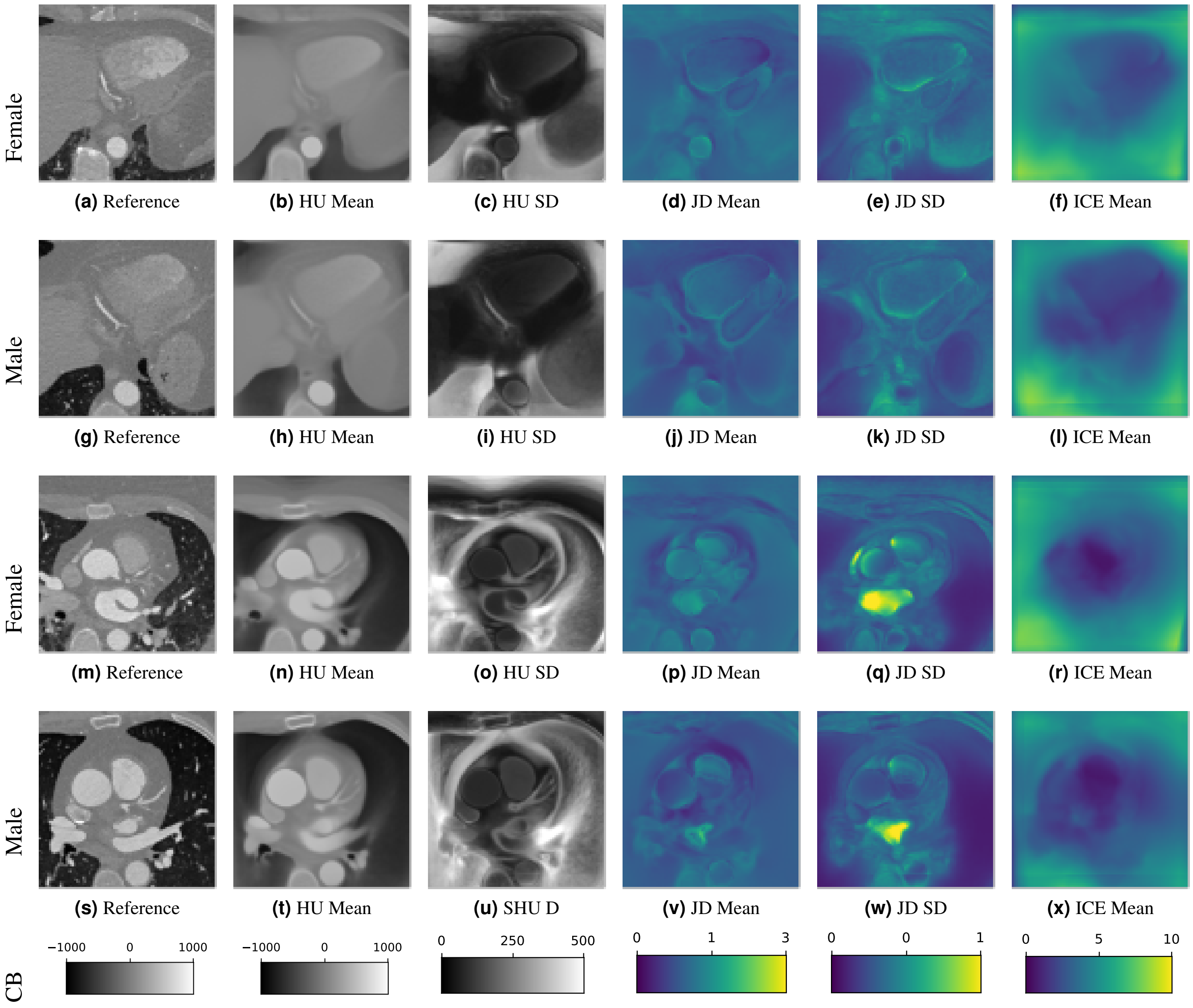

Figure S-5. Additional visualizations of the registration performance through example axial slices.

C Sensitivity Analysis: Dice-filtered Imiomics Maps

In Fig. S-6 we show the imiomics maps after filtering out severe outliers with low registration performance, as measured with the Dice coefficient of the 5 major segmented structures.

Supervoxel-wise association with Dice coefficient filtering sensitivity analysis

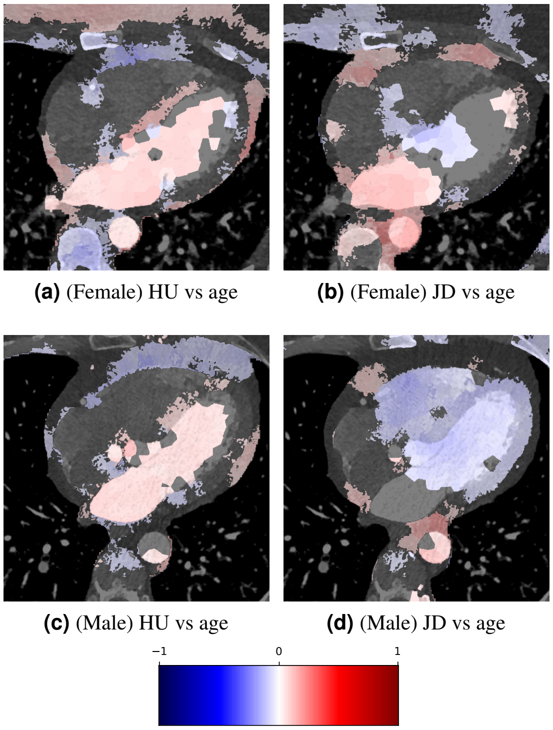

(a) (Female) HU vs age

(b) (Female) JD vs age

(c) (Male) HU vs age

(d) (Male) JD vs age

Figure S-6. Sensitivity analysis of the supervoxel-wise analysis of selected axial slices of attenuation, and local volume/JacDet for the female and male cohorts with Dice coefficient filtering enabled, excluding subjects with a Dice coefficient of less than 0.7 in any of the 5 evaluated structures (LV, LA, RV, RA, Myo). Each image is displayed with the correlation map overlaid on the reference image, where absence of color means non-significance.
